# Immune recovery markers in a double blind clinical trial comparing dolutegravir and raltegravir based regimens as initial therapy (SPRING-2)

**DOI:** 10.1371/journal.pone.0226724

**Published:** 2020-01-16

**Authors:** Jose-Ramon Blanco, Belen Alejos, Santiago Moreno

**Affiliations:** 1 Department of Infectious Diseases, Hospital San Pedro–CIBIR, Logroño (La Rioja), Spain; 2 Centro Nacional de Epidemiología, Instituto de Salud Carlos III, Madrid, Spain; 3 Department of Infectious Diseases, Hospital Ramón y Cajal, Alcalá de Henares University, Instituto Ramón y Cajal de Investigación Sanitaria (IRYCIS), Madrid, Spain; Imperial College London, UNITED KINGDOM

## Abstract

**Background:**

Multiple T-cell marker recovery (MTMR: CD4+ T-cells >500 cel/mm^3^ plus CD4+% >29% plus CD4+/CD8+ ratio >1) has been proposed as the most complete level of immune reconstitution. In this study we quantified differences in the CD4+/CD8+ ratio, CD4+% recovery and MTMR after starting HIV-1 treatment with dolutegravir (DTG) vs. raltegravir (RAL) plus a NRTI backbone.

**Methods:**

Exploratory post-hoc analysis of the SPRING-2 study, a randomized double-blind clinical trial comparing DTG and RAL as third agents in naive HIV-infected patients at 100 sites in Canada, USA, Australia, and Europe. Percentage differences and corresponding precision based on 95% confidence intervals (CI) and p-values were calculated for i) CD4+/CD8+ ratio normalization, ii) CD4+% normalization, and iii) the achievement of MTMR.

**Results:**

A total of 822 participants were analyzed (411 in each group). No statistically significant differences in the proportion of patients who reached a CD4+/CD8+ ratio ≥0.5 & ≥1 at w48 & w96 were observed. At w96, the proportion of patients with a CD4+/CD8+ ratio ≥1 was similar (30.43% DTG vs. 29.57% RAL). No differences were observed in the mean increase in CD4+/CD8+ ratio from baseline at both w48 & w96. Similarly, no significant differences in the CD4+/CD8+>29% were observed at w96 (72.95% DTG vs 69.28% RAL). The proportion of patients attaining MTMR criteria was also similar in the DTG group and the RAL group at w48 (20.33% vs. 18.26%; difference 2.07 (95%CI (-3.67;7.81) P = 0.481 and w96 (28.70% vs. 27.13; difference 1.56 (95%CI -5.22;8.34) P = 0.652).

**Conclusion:**

After comparing DTG and RAL, no differences on immune recovery markers were observed.

## Introduction

Despite sustained virological suppression, complete recovery of the immune system is difficult to achieve with antiretroviral therapy [1, 2]. Besides the absolute CD4 T-cell count, the CD4+/CD8+ ratio [3–9] and the CD4 T-cell percentage (CD4+%) predicts the risk of AIDS and non-AIDS events [10–13]. Although a single marker may be easier to use, combinations could provide more robust information regarding the immune system restoration, and this is the basis for the recommendation to use, as well, the multiple T-cell marker recovery (MTMR) (CD4+ T-cells >500/mm^3^ plus CD4+% >29% plus CD4+/CD8+ ratio >1) [14].

The integrase strand transfer inhibitors (INSTIs) are widely used antiretroviral drugs and are currently recommended as the drugs of choice in the initial therapy against HIV-infection [15, 16]. Regimens that include INSTI have a high efficacy and good tolerability, and achieves HIV-1 RNA viral suppression faster than regimens that contain protease inhibitors [17, 18] or nonnucleoside reverse transcriptase inhibitors (NNRTIs) [19, 20]. So far, only one study (SPRING-2 study) has compared two drugs in this class, raltegravir (RAL) and dolutegravir (DTG). This study showed that time for achieving virological control was similar with the two drugs, as well as the proportion of patients who achieved virological control and the gain in CD4+ T-cell count [21].

The SPRING-2 study offers the opportunity to examine the effect of two INSTI on markers of immune restoration beyond the CD4 T cell count. We have conducted this study to quantify the differences in the CD4+/CD8+ ratio and the CD4+% recovery and to determine the percentage of patients who achieve MTMR [14] after starting treatment with either DTG or RAL. This study drugs were given with coformulated tenofovir/emtricitabine (TDF/FTC) or abacavir/lamivudine (ABC/3TC).

## Materials and methods

The SPRING-2 (ING113086) study was a randomised, double-blind, active-controlled, double placebo, multicentre, parallel-group, non-inferiority study. Adults (aged ≥18 years) naive for antiretroviral therapy with HIV-1 infection and HIV-1 RNA of 1000 copies per mL or more were recruited from 100 sites in Australia, Europe, Canada, and the USA. Study methods and eligibility criteria have been published previously [21]. The study was designed to assess the efficacy and safety of DTG versus RAL, in combination with two widely recommended NRTI backbones, as first-line treatment for antiretroviral-naive adults with HIV-1. At the investigators’ discretion, patients received an NRTI backbone of coformulated TDF/FTC or ABC/3TC. The primary analysis occurred at week 48. Efficacy and safety analyses were performed in the intention-to-treat population and safety population, respectively; both populations included all participants who underwent randomization and received at least one dose of the study drugs. Baseline demographics and disease characteristics were similar between treatment groups. Visits were scheduled at baseline and at weeks 2, 4, 8, 12, 16, 24, 32, 40, and 48. After the week 48 visit, participants continued to receive blinded treatment until week 96, with visits scheduled every 12 weeks.

We have performed an independent exploratory post-hoc analysis in the full SPRING-2 study, not included in the pre-registered analysis plan. Ethics committee approval was obtained at all participating centres in accordance with the principles of the 2008 Declaration of Helsinki ((https://clinicaltrials.gov/ct2/show/study/NCT01227824?show_locs=Y#locn). Competent Authority or Ethics Committee in each country concerned was obtained. Each patient gave written informed consent before undergoing study procedures. This trial is registered with ClinicalTrials.gov, number NCT01227824 and in the EudraCT, number2009-017950-11. Authors had no access to any identifying participant information and accessed the data via Clinical study Data Request (CSDR). CSDR is a data sharing community that facilitates access to patient-level data from clinical studies (https://clinicalstudydatarequest.com/Default.aspx).

Based on the criteria described previously [22], we assessed three different primary immunological outcomes: i) the achievement of CD4+/CD8+ ratio normalization at cut-offs of 0.5 and 1, ii) the achievement of CD4+% normalization at a cut-off of 29%, and iii) the achievement of MTMR.

An exploratory post hoc analysis was performed in the intention-to-treat population. The primary approach for handling missing data was the observed-cases approach. In this approach, only cases with available data for a particular time point are included, which enables evaluation of immunological normalization without confounding by discontinuations or lack of observation. Balance in the treatment group for the main baseline covariates was assessed using the non-parametric Mann-Whitney U test or the chi-squared test as appropriate.

Percentage differences (DTG vs. RAL) and corresponding precision based on 95% confidence intervals (CI) and p-values were calculated for i) CD4+/CD8+ ratio normalization (≥0.5 and ≥1), ii) the achievement of CD4+% normalization (≥29%), and iii) the achievement of MTMR at 48 and 96 weeks. General estimating equations (GEEs) with logit link function were used to estimate odds ratios (ORs) for the impact of treatment group on the immunological outcomes previously described.

To evaluate the impact of the cut-off selection, we also used a repeated-measures mixed models that included the interaction between treatment and study visit, to calculate the mean changes and 95% CI by treatment arm as well as the estimated mean differences (95% CI) in the mean changes. No assumptions were made about the correlations among the various measurements of a participant (i.e., the correlation matrix for within participant errors is unstructured).

A sensitivity analysis was performed considering time to CD4+/CD8+ normalization, to CD4+% normalization and to achievement of MTMR as endpoints. We used the multiple decrement method to calculate the cumulative incidence [23] of the endpoints and a proportional hazards model on the sub-distribution Hazard [24, 25] to estimate sub-Hazard Ratios (sHR) for the effect treatment group, treating deaths prior to the endpoint of interest as competing events in every model.

We performed multivariable regression models to analyse the impact of DTG and RAL on the immunological outcomes previously described after adjustment for the following potential confounders: baseline CD4, baseline CD4+%, baseline CD8, baseline CD4+/CD8+, baseline viral load, backbone dual NRTI, HIV risk category, age and sex. We performed the multivariable regression analyses in two stages; in the first stage, the baseline value of each primary immunological outcome entered into the regression. In the second stage, the rest of baseline covariates were added to the adjusted model. Multicollinearity between confounding factors in the final model was evaluated using the variance inflation factor (VIF).

As a complementary analysis, we calculated the proportion of patients attaining CD4+/CD8+ ratio normalization, CD4+% normalization and MTMR stratified by CD4 count and HIV viral load at baseline.

All statistical analyses were performed using Stata software (version 15.0; College Station, TX).

## Results

A total of 822 participants were randomized and received at least one dose of study medication (411 in each group). Demographic and disease characteristics at baseline were well balanced across treatment groups and were presented previously [21]. Patients predominantly had HIV-1 subtype B, with A1 being the next most common and 60% received TDF/FTC as NRTI backbone. Median age was 36 years (18–75), and there was a high representation of men (86%) and of white individuals (85%). HIV-RNA was >100,000 copies/mL in 32% of the subjects, and the CD4+ T-cell count was <350 cells/mm^3^ in 47%. Median percentage and absolute CD4+ T cell count was 22% (16–27) and 361 cells/mm^3^ (271–459), respectively. Median CD4+/CD8+ ratio was 0.38 (0.27–0.54). Other baseline immunologic characteristics are presented in Table 1. Differences by treatment group were observed in the proportion of patients with MTMR (CD4+ T cells >500/mm^3^ plus CD4+% 29% plus CD4+/CD8+ ratio >1).

**Table 1:** Baseline clinical, virological and immunologic characteristics.

	DOLUTEGRAVIR	RALTEGRAVIR	TOTAL	P
	n (%)	n (%)	n (%)	
TOTAL	411	411	822	
**CDC category**				0.433
Category A	359 (87%)	347 (84%)	706 (86%)	
Category B	43 (10%)	55 (13%)	98 (12%)	
Category C	9 (2%)	9 (2%)	18 (2%)	
**Viral Load (c/mL)**				0.877
< = 100,000	297 (72%)	295 (72%)	295 (36%)	
>100,000	114 (28%)	116 (28%)	116 (14%)	
**CD4+ (cells/mm^3)**				0.931
<50	144 (35%)	139 (34%)	283 (34%)	
50 to <200	126 (31%)	136 (33%)	262 (32%)	
200 to <350	47 (11%)	44 (11%)	91 (11%)	
350 to <500	8 (2%)	6 (1%)	14 (2%)	
> = 500	86 (21%)	86 (21%)	172 (21%)	
**CD4+/CD8+ ratio**				0.179
<0.5	281 (68%)	278 (68%)	559 (68%)	
0.5–1	105 (26%)	120 (29%)	225 (27%)	
> = 1	14 (3%)	8 (2%)	22 (3%)	
Unknown	11 (3%)	5 (1%)	16 (2%)	
**% CD4+**				0.863
<29%	326 (79%)	328 (80%)	654 (80%)	
> = 29%	85 (21%)	83 (20%)	168 (20%)	
**MTMR**				0.012
No MTMR	387 (94%)	403 (98%)	790 (96%)	
MTMR	13 (3%)	3 (1%)	16 (2%)	
Unknown	11 (3%)	5 (1%)	16 (2%)	
	**Median (Interquartile Range)**			
**CD4+ (cells/mm^3)**	359 (276–470)	362 (267–469)	361 (271–469)	0.846
**% CD4+**	21 (16–27)	22 (17–27)	22 (16–27)	0.623
CD8+ (cells/mm^3)*	900 (670–1253)	921 (659–1221)	901 (665–1235)	0.947
CD4+/+CD8*	0.37 (0.27–0.54)	0.39 (0.26–0.53)	0.38 (0.27–0.54)	0.611

*Baseline values available for 400 in DOLUTEGRAVIR and 406 RALTEGRAVIR groups

Discontinuations due to other reasons while HIV-1 RNA not <50 copies per mL were higher in the RAL arm, whereas the numbers of participants that discontinued due to protocol deviation, lost to follow-up, withdrawal of consent or were excluded at the investigators’ discretion were similar between arms, as previously described [21]. The proportion of subjects with missing data during the window used for endpoint calculations was similar in both treatment arms. Median CD4 cell counts increased in both treatment groups from baseline to week 48 [230 cells/mm^3^ (IQR: 128–338) in the DTG group, 230 cells/mm^3^ (IQR: 139–354) in the RAL group] and to week 96 [274 cells/mm^3^ (IQR: 159–399) and 264 cells/mm^3^ (IQR: 151–396) respectively].

**Fig 1:**
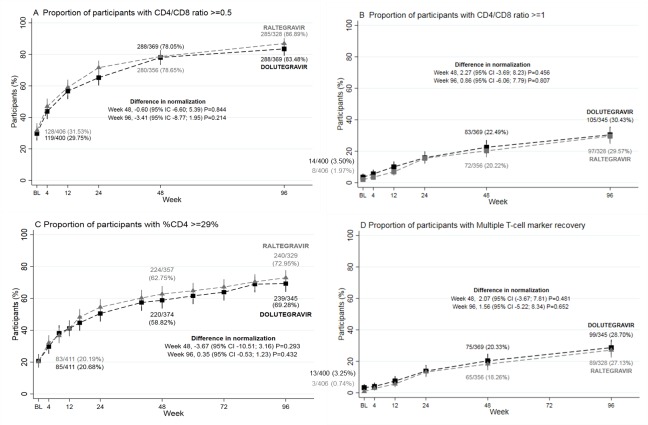
CD4+/CD8+ ratio normalization, CD4+ percentage normalization and Multiple T-cell marker recovery (MTMR: CD4+ T cells >500/mm3 plus CD4+% 29% plus CD4+/CD8+ ratio >1) differences by treatment group.

The proportions of patients attaining CD4+/CD8+ ratio normalization with DTG and RAL at the cut-offs of 0.5 and 1, CD4+% normalization and MTMR are shown in **Fig 1**. There were no statistically significant differences in the proportion of patients who reached a CD4+/CD8+ ratio ≥0.5 and ≥1 at weeks 48 and 96. At week 96, the proportion of patients with a CD4+/CD8+ ratio ≥1 was 30.43% in the DTG group and 29.57% in the RAL group [difference 0.86 95% IC (-6.06; 7.79); p = 0.807]; adjusted Odds Ratio (aOR) 1.106 95% IC (0.655; 1.867) p = 0.970]. The mean increase in CD4+/CD8+ ratio from baseline was also similar in the DTG arm and in the RAL arm at both week 48 (0.35 vs. 0.35; adjusted difference -0.001 95% CI (-0.031; 0.029) p = 0.947) and week 96 (0.45 vs. 0.44; adjusted difference 0.007 95% IC (-0.023; 0.037) p = 0.645).

No significant differences in the proportion of patients attaining CD4+% normalization by treatment group were observed during the study; at week 96, the proportion of patients with a CD4+% ≥0.29 was 72.95% in the DTG group and 69.28% in the RAL group (difference 0.35 95% IC (-5.3; 1.23); p = 0.432); adjusted Odds Ratio (aOR) 1.044 95% IC (0.628; 1.736) p = 0.868). Mean CD4+ percentage increased from baseline to week 48 (increase of 8.74 for DTG and 8.75 for RAL; adjusted difference -0.031 95% CI (-0.664; 0.602); p = 0.924) and to week 96 (11.15 vs.10.80; adjusted difference 0.394 95% CI (-0.257; 1.044); p = 235).

The proportion of patients attaining a CD4 T-cell count >500/mm^3^ plus a CD4+% >29% plus a CD4+/CD8+ ratio >1 was also similar in the DTG group and the RAL group at week 48 (20.33% vs. 18.26%; difference 2.07 (95% CI (-3.67; 7.81) P = 0.481 and week 96 (28.70% vs. 27.13; difference 1.56 (95% CI -5.22; 8.34) P = 0.652).

**Fig 2:**
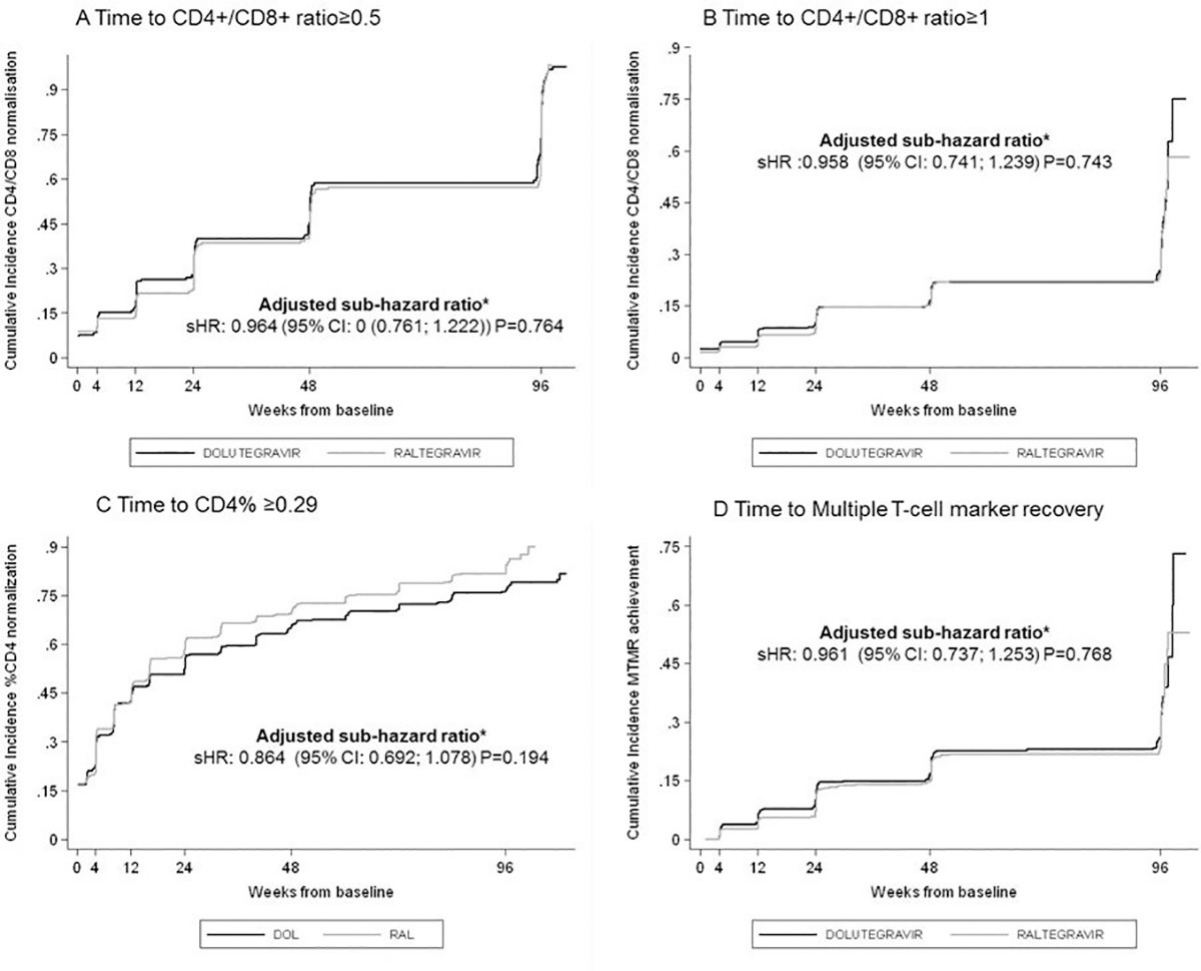
Cumulative Incidence Plot of Time to CD4+/CD8+ ratio normalization, CD4+ percentage normalization and Multiple T-cell marker recovery (MTMR: CD4+ T cells >500/mm3 plus CD4+% 29% plus CD4+/CD8+ ratio >1). sHR sub hazard ratio (efavirenz/tenofovir/emtricitabine compared to dolutegravir/abacavir/lamivudine); *Adjusted by baseline CD4+/CD8+, baseline %CD4, baseline CD4, baseline CD8 baseline Viral Load, backbone dual NRTI, HIV risk category, age and sex.

The sensitivity analysis including the time to normalization of the CD4+/CD8+ ratio, CD4+% and MTMR produced results that were concordant with the other approaches (**Fig 2**). We observed a longer time to CD4+% normalization in the DTG group compared to the RAL group nevertheless these differences were not significant after adjusting for potential confounders (adjusted sHR 0.864 95% CI (0.692; 1.078) p = 0.194).

Results from crude and multivariable regression models are showed in **S1–S3 Tables.**

**Fig 3:**
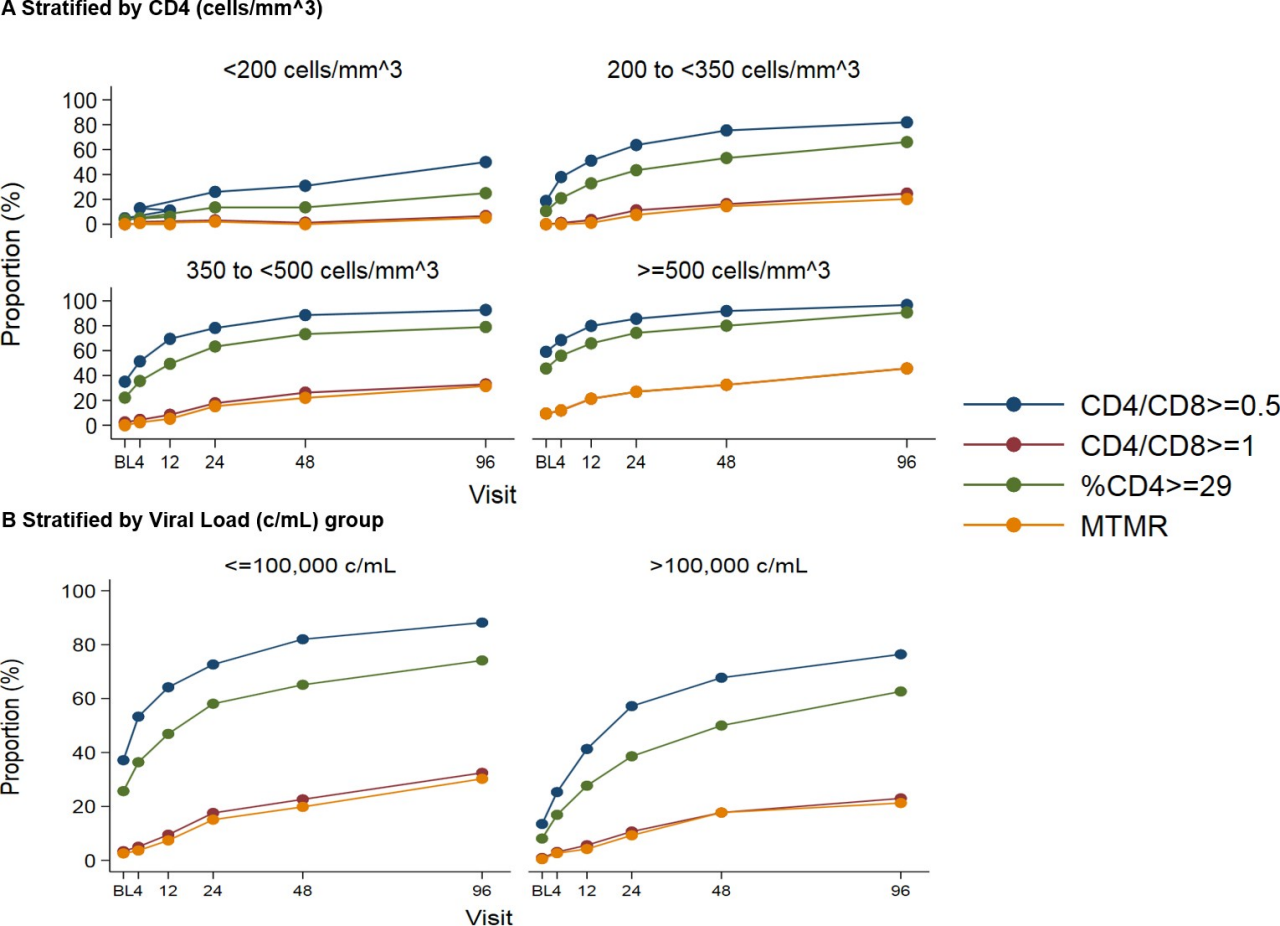
CD4+/CD8+ ratio normalization, CD4+ percentage normalization and Multiple T-cell marker recovery (MTMR: CD4+ T cells >500/mm3 plus CD4+% 29% plus CD4+/CD8+ ratio >1) differences by CD4 count and HIV Viral Load at baseline.

Finally, **Fig 3** shows the proportion of patients attaining CD4+/CD8+ ratio normalization, CD4+% normalization and MTMR stratified by CD4 count and HIV viral load at baseline by visit. Increases in the different primary immunological outcomes occurred up to 96 weeks after starting ART. The proportion of individuals with normalized immunological markers was higher among patients with higher CD4 counts and lower HIV-RNA at baseline.

## Discussion

Our analysis of the information collected in the SPRING-2 study failed to show any difference in immune recovery markers between RAL and DTG. The study showed that more than 78% of patients treated with DTG or RAL achieved a CD4+/CD8+ ratio ≥0.5 by week 48, and this percentage continued increasing through week 96 (higher than 83% for both therapeutic options). Regarding the CD4+/CD8+ ratio cut-off of ≥1.0, more than 20% of patients treated with any therapeutic option were above the cut-off by week 48, and this percentage also continued to increase through week 96.

We and other authors have evaluated the impact of different antiretroviral drugs on immune recovery markers. RAL has been associated with an increased CD4+/CD8+ ratio in two studies where patients were switched or intensified with this drug [26, 27]. In an analysis of the SINGLE study, which compared DTG and the NNRTIs, efavirenz (EFV), we could show similar findings [22] but, at week 96, the proportion of patients with a CD4+/CD8+ ratio ≥1 was higher in the EFV group [difference 11.70 95% IC (4.49; 18.91); P = 0.002]. In that study the improvement of the CD4+/CD8+ ratio ≥1 at week 96 could not be attributed to the nucleosides in the backbone, the third drug, a better virological efficacy, or a higher CD4 cell recovery [22].

Serrano-Villar et al [28] analysed the CD4+/CD8+ ratio in the STARMRK study, a double-blind randomised controlled trial of RAL-based vs. EFV-based combination therapy in treatment-naïve patients, and found that RAL was associated with higher rates of CD4+/CD8+ ratio normalization at a cut-off of >0.4. This finding was not reproduced for other CD4+/CD8+ ratio cut-offs (>1, >1.5, and >2.0). Unlike our analysis, the authors employed a different cut-off (0.4) and employed a linear mixed model to define the CD4+/CD8+ ratio response.

Although the clinical and prognosis implications of CD4+/CD8+ ratio are still controversial, it is agreed that the higher, the better. Some authors have observed that a lower CD4+/CD8+ ratio in HIV-infected patients was associated with an increased risk of AIDS events and AIDS-related death [5], but a recent observational cohort of virologically suppressed patients observed a little evidence for non-AIDS mortality [29]. On the other hand, a CD4+/CD8+ ratio <1 in seronegative patients has been associated with immunosenescence and mortality [30–32]. Unfortunately, Mutoh et al. [33] reported the lack of normalization CD4+/CD8+ ratio (adjusted mean values, 0.89) compared to the levels seen in healthy individuals even after long-term successful ART in patients with suppressed viral load.

CD4+% has been proposed as another independent predictive factor of AIDS progression [11]. In this study, no differences were observed after comparing DTG and RAL neither at week 48 or week 96. To date, only a post-hoc analysis of the SINGLE study has evaluated this marker [22], with results similar to those reported in the present report. Mutoh et al [33] did not observe, in patients with an undetectable viral load after long-term successful ART, the recovery of the CD4+% (adjusted mean values, 29.5%) to the values observed in seronegative population. Its clinical implications are unknown.

In the same way, MTMR could be a robust predictor of immune recovery because reconstitution of absolute CD4 T-cell counts does not always reflect normalization of T-cell homeostasis [14]. In our study, MTMR was also similar with DTG and RAL. Significant differences were neither found between RAL and EFV in a post-hoc analysis of the SINGLE study [22].

Our study has some limitations. First, we did not analyse variables related to reduced immunological recovery such as cytomegalovirus serology [5, 34], but the double blind design of the study limits any bias in this sense. Second, this is a post-hoc analysis. However, these analyses are common in large multicentre clinical trials showing the utility and the development of data registries [22, 28, 35, 36]. Indeed, post-hoc analyses of data can generate scientific hypotheses that could be studied in future randomized studies and explores unanticipated gaps in study design [37]. On the contrary, this study has important strengths such as its methodology (a randomized, double-blind clinical trial) that has made a several contributions in well-known and influential journals [21, 38].

In conclusion, our study, that compares for the first time two INSTIs in terms of their impact on CD4+/CD8+ ratio, CD4+% and MTMR recovery, shows no differences between the drugs in any of the parameters analysed. Given the potential clinical significance of immunology recovery, the CD4+/CD8+ ratio and other parameters should be included in clinical trials that evaluate new antiretroviral drugs.

## Supporting information

S1 Table Crude and adjusted Odds Ratios (OR) for CD4/CD8 normalization, mean differences in CD4/CD8 changes from baseline and sub-distribution hazard ratios (sHR) for time to CD4/CD8 normalization. *Adjusted by baseline CD4/CD8, **Adjusted by baseline CD4/CD8, baseline %CD4, baseline CD4, baseline CD8 baseline Viral Load, backbone dual NRTI, HIV risk category, age and sex.
(DOCX)

S2 Table Crude and adjusted Odds Ratios (OR) for %CD4 normalization, mean differences in %CD4 changes from baseline and sub-distribution hazard ratios (sHR) for time to %CD4 normalization. * Adjusted by baseline %CD4, **Adjusted by baseline %CD4, baseline CD4/CD8, baseline CD4, baseline CD8 baseline Viral Load, backbone dual NRTI, HIV risk category, age and sex.
(DOCX)

S3 Table Crude and adjusted Odds Ratios (OR) for multiple T-cell marker recovery (MTMR: CD4+ T cells >500/mm^3^ plus CD4+% >29% plus CD4+/CD8+ ratio >1) and sub-distribution hazard ratios (sHR) for time to MTMR. * Adjusted by baseline %CD4, baseline CD4/CD8, baseline CD4, **Adjusted by baseline %CD4, baseline CD4/CD8, baseline CD4, baseline CD8 baseline Viral Load, backbone dual NRTI, HIV risk category, age and sex.
(DOCX)
