# Supplementary material for: Immune recovery markers in a double blind clinical trial comparing dolutegravir and raltegravir based regimens as initial therapy (SPRING-2)
Source: PLoS One. 2020 Jan 16;15(1):e0226724. doi: 10.1371/journal.pone.0226724 (PMC6964875; doi:10.1371/journal.pone.0226724)
Supplement: S1 Table — *Adjusted by baseline CD4/CD8, **Adjusted by baseline CD4/CD8, baseline %CD4, baseline CD4, baseline CD8 baseline Viral Load, backbone dual NRTI, HIV risk category, age and sex. (DOCX) [file pone.0226724.s001.docx]

**S1 Table: Crude and adjusted Odds Ratios (OR) for CD4/CD8 normalization, mean differences in CD4/CD8 changes from baseline and sub-distribution hazard ratios (sHR) for time to CD4/CD8 normalization.**

|  |  | | **Crude** | | **Adjusted for baseline*** | | **Adjusted for confounders**** | |
| --- | --- | --- | --- | --- | --- | --- | --- | --- |
| **CD4/CD8 normalization** | | | **OR (95% IC)** | **P** | **OR (95% IC)** | **P** | **OR (95% IC)** | **P** |
| **CD4/CD8 ≥0.5** | | |  |  |  |  |  |  |
| **Week 48** | | RALTEGRAVIR | 1 |  | 1 |  | 1 |  |
|  |  | DOLUTEGRAVIR | 1.009 (0.718; 1.417) | 0.961 | 1.092 (0.643; 1.854) | 0.745 | 1.106 (0.655; 1.867) | 0.707 |
| **Week 96** | | RALTEGRAVIR | 1 |  | 1 |  | 1 |  |
|  |  | DOLUTEGRAVIR | 0.799 (0.531; 1.202) | 0.282 | 0.758 (0.421; 1.364) | 0.355 | 0.767 (0.430; 1.365) | 0.367 |
| **CD4/CD8 ≥1** | | |  |  |  |  |  |  |
| **Week 48** | | RALTEGRAVIR | 1 |  | 1 |  | 1 |  |
|  |  | DOLUTEGRAVIR | 1.105 (0.776; 1.574) | 0.581 | 1.105 (0.709; 1.723) | 0.660 | 1.103 (0.701; 1.735) | 0.672 |
| **Week 96** | | RALTEGRAVIR | 1 |  | 1 |  | 1 |  |
|  |  | DOLUTEGRAVIR | 0.969 (0.701; 1.340) | 0.850 | 1.009 (0.665; 1.530) | 0.967 | 0.996 (0.652; 1.522) | 0.985 |
| **CD4/CD8 change from baseline** | | | **Mean diff (95% IC)** | **p** | **Mean diff (95% IC)** | **p** | **Mean diff (95% IC)** | **P** |
| **Week 48** | | RALTEGRAVIR | 0 |  | 0 |  | 0 |  |
|  |  | DOLUTEGRAVIR | 0.001 (-0.029; 0.031) | 0.960 | 0.000 (-0.030; 0.030) | 0.983 | -0.001 (-0.031; 0.029) | 0.947 |
| **Week 96** | | RALTEGRAVIR | 0 |  | 0 |  | 0 |  |
|  |  | DOLUTEGRAVIR | 0.009 (-0.022; 0.039) | 0.577 | 0.008 (-0.022; 0.039) | 0.595 | 0.007 (-0.023; 0.037) | 0.645 |
| **Time to CD4/CD8 normalization** | | | **sHR (95% IC)** | **P** | **sHR (95% IC)** | **P** | **sHR (95% IC)** | **P** |
| **CD4/CD8 ≥0.5** | | |  |  |  |  |  |  |
| RALTEGRAVIR | | | 1 |  | 1 |  | 1 |  |
| DOLUTEGRAVIR | | | 1.096 (0.895; 1.343) | 0.373 | 0.991 (0.784; 1.253) | 0.942 | 0.964 (0.761; 1.222) | 0.764 |
| **CD4/CD8 ≥1** | | |  |  |  |  |  |  |
| RALTEGRAVIR | | | 1 |  | 1 |  | 1 |  |
| DOLUTEGRAVIR | | | 1.022 (0.804; 1.299) | 0.860 | 0.951 (0.723; 1.251) | 0.720 | 0.958 (0.741; 1.239) | 0.743 |

** Adjusted by baseline CD4/CD8,*

***Adjusted by baseline CD4/CD8, baseline %CD4, baseline CD4, baseline CD8 baseline Viral Load, backbone dual NRTI, HIV risk category, age and sex*
