# Supplementary material for: Immune recovery markers in a double blind clinical trial comparing dolutegravir and raltegravir based regimens as initial therapy (SPRING-2)
Source: PLoS One. 2020 Jan 16;15(1):e0226724. doi: 10.1371/journal.pone.0226724 (PMC6964875; doi:10.1371/journal.pone.0226724)
Supplement: S2 Table — * Adjusted by baseline %CD4, **Adjusted by baseline %CD4, baseline CD4/CD8, baseline CD4, baseline CD8 baseline Viral Load, backbone dual NRTI, HIV risk category, age and sex. (DOCX) [file pone.0226724.s002.docx]

**S2 Table: Crude and adjusted Odds Ratios (OR) for %CD4 normalization, mean differences in %CD4 changes from baseline and sub-distribution hazard ratios (sHR) for time to %CD4 normalization.**

|  |  | **Crude** | | **Adjusted for baseline*** | | **Adjusted for confounders**** | |
| --- | --- | --- | --- | --- | --- | --- | --- |
| **% CD4 normalization (>=29)** | | **OR (95% IC)** | **P** | **OR (95% IC)** | **P** | **OR (95% IC)** | **P** |
| **Week 48** | RALTEGRAVIR | 1 |  | 1 |  | 1 |  |
|  | DOLUTEGRAVIR | 0.884 (0.662; 1.179) | 0.400 | 0.961 (0.604; 1.528) | 0.866 | 0.951 (0.596; 1.519) | 0.834 |
| **Week 96** | RALTEGRAVIR | 1 |  | 1 |  | 1 |  |
|  | DOLUTEGRAVIR | 0.882 (0.641; 1.212) | 0.438 | 1.030 (0.622; 1.706) | 0.907 | 1.044 (0.628; 1.736) | 0.868 |
| **% CD4 change from baseline** | | **Mean diff (95% IC)** | **p** | **Mean diff (95% IC)** | **p** | **Mean diff (95% IC)** | **P** |
| **Week 48** | RALTEGRAVIR | 0 |  | 0 |  | 0 |  |
|  | DOLUTEGRAVIR | -0.060 (-0.736; 0.616) | 0.862 | -0.069 (-0.726; 0.588) | 0.837 | -0.031 (-0.664; 0.602) | 0.924 |
| **Week 96** | RALTEGRAVIR | 0 |  | 0 |  | 0 |  |
|  | DOLUTEGRAVIR | 0.243 (-0.448; 0.934) | 0.490 | 0.235 (-0.437; 0.907) | 0.493 | 0.394 (-0.257; 1.044) | 0.235 |
| **Time to %CD4 normalization (>=29)** | | **sHR (95% IC)** | **P** | **sHR (95% IC)** | **P** | **sHR (95% IC)** | **P** |
| RALTEGRAVIR | | 1 |  | 1 |  | 1 |  |
| DOLUTEGRAVIR | | 0.855 (0.736; 0.993) | 0.040 | 0.890 (0.723; 1.094) | 0.267 | 0.864 (0.692; 1.078) | 0.194 |

** Adjusted by baseline %CD4,*

***Adjusted by baseline %CD4, baseline CD4/CD8, baseline CD4, baseline CD8 baseline Viral Load, backbone dual NRTI, HIV risk category, age and sex*
