# Supplementary material for: Immune recovery markers in a double blind clinical trial comparing dolutegravir and raltegravir based regimens as initial therapy (SPRING-2)
Source: PLoS One. 2020 Jan 16;15(1):e0226724. doi: 10.1371/journal.pone.0226724 (PMC6964875; doi:10.1371/journal.pone.0226724)
Supplement: S3 Table — * Adjusted by baseline %CD4, baseline CD4/CD8, baseline CD4, **Adjusted by baseline %CD4, baseline CD4/CD8, baseline CD4, baseline CD8 baseline Viral Load, backbone dual NRTI, HIV risk category, age and sex. (DOCX) [file pone.0226724.s003.docx]

**S3 Table: Crude and adjusted Odds Ratios (OR) for multiple T-cell marker recovery (MTMR: CD4+ T cells >500/mm^3^ plus CD4+%** >**29% plus CD4+/CD8+ ratio** >**1) and sub-distribution hazard ratios (sHR) for time to MTMR.**

|  |  | **Crude** | | **Adjusted for baseline*** | | **Adjusted for confounders**** | |
| --- | --- | --- | --- | --- | --- | --- | --- |
| **MTMR Achievement** | | **OR (95% IC)** | **P** | **OR (95% IC)** | **P** | **OR (95% IC)** | **P** |
| **Week 48** | RALTEGRAVIR | 1 |  | 1 |  | 1 |  |
|  | DOLUTEGRAVIR | 1.076 (0.744; 1.554) | 0.698 | 1.037 (0.658; 1.635) | 0.877 | 1.048 (0.659; 1.669) | 0.842 |
| **Week 96** | RALTEGRAVIR | 1 |  | 1 |  | 1 |  |
|  | DOLUTEGRAVIR | 1.007 (0.723; 1.402) | 0.967 | 1.028 (0.682; 1.551) | 0.894 | 1.031 (0.676; 1.572) | 0.888 |
| **Time to MTMR Achievement** | | **sHR (95% IC)** | **P** | **sHR (95% IC)** | **P** | **sHR (95% IC)** | **P** |
| RALTEGRAVIR | | 1 |  | 1 |  | 1 |  |
| DOLUTEGRAVIR | | 1.048 (0.817; 1.343) | 0.713 | 0.972 (0.749; 1.262) | 0.831 | 0.961 (0.737; 1.253) | 0.768 |

** Adjusted by baseline %CD4, baseline CD4/CD8, baseline CD4*

***Adjusted by baseline %CD4, baseline CD4/CD8, baseline CD4, baseline CD8 baseline Viral Load, backbone dual NRTI, HIV risk category, age and sex*
